# Characteristics and chemical compositions of propolis from Ethiopia

**DOI:** 10.1186/2193-1801-3-253

**Published:** 2014-05-20

**Authors:** Ahmed I Rushdi, Nuru Adgaba, Noofal I M Bayaqoob, Ahmed Al-Khazim, Bernd I T Simoneit, Aarif H El-Mubarak, Khalid F Al-Mutlaq

**Affiliations:** Chair of Green Energy Research, College of Food and Agriculture Sciences, King Saud University, P. O. Box 2460, Riyadh, 11451 Saudi Arabia; College of Earth, Oceanic and Atmospheric Sciences, Oregon State University, Corvallis, Oregon 97331 USA; Department of Earth and Environmental Sciences, Faculty of Science, Sana’a University, Sana’a, Yemen; Bee Research Unit, Plant Protection Department, College of Food and Agriculture Sciences, King Saud University, P. O. Box 2460, Riyadh, 11451 Saudi Arabia; Department of Chemistry, Oregon State University, Corvallis, Oregon 97331 USA

**Keywords:** Propolis, Ethiopia, Triterpenoids, GC-MS

## Abstract

**Introduction:**

Propolis is a sticky material mixed by honeybees to utilize it in protecting their hives from infection by bacteria and fungi. The therapeutic properties of propolis are due to its chemical composition with bio-active compounds; therefore, researchers are interested in studying its chemical constituents and biological properties. The main objective of this study is to determine the chemical compositions, characteristics and relative concentrations of organic compounds in the extractable organic matter of propolis samples collected from four different areas in Ethiopia.

**Results:**

The propolis samples were extracted with a mixture of dichloromethane and methanol and analyzed by gas chromatography–mass spectrometry (GC-MS).The results showed that the total extract yields ranged from 27.2% to 64.2% (46.7 ± 19.1%). The major compounds were triterpenoids (85.5 ± 15.0% of the total extracts, mainly α-, β-amyrins and amyryl acetates), *n*-alkanes (5.8 ± 7.5%), *n*-alkenes (6.2 ± 7.0%,), methyl *n*-alkanoates (0.4 ± 0.2%), and long chain wax esters (0.3 to 2.1%).

**Conclusion:**

The chemical compositions of these propolis samples indicate that they are potential sources of natural bio-active compounds for biological and pharmacological applications.

## Introduction

Honeybees collect resinous/waxy substances from exudates of plants to make a sticky material known as propolis (Ghisalberti 1979; Parolia et al. 2010). They utilize propolis to seal cracks in hives, encapsulate invader carcasses and protect their hives from infection by bacteria and fungi (Banskota et al. 2001; Simone-Finstrom & Spivak 2010). In ancient times, Egyptians, Greeks and Romans all used propolis as a remedy against some diseases (Sforcin & Bankova 2011). The therapeutic properties of propolis are due to its chemical composition with bio-active compounds; therefore, researchers are interested in studying its chemical constituents and biological properties (Sforcin & Bankova 2011; Bankova 2005; Castaldo & Capasso 2002; Sforcin 2007). The diverse chemical compositions and biological activities of propolis are attributed to geographical settings, plant sources and collecting season (Sforcin & Bankova 2011). Flavonoids, aromatic acids, diterpenoid acids, triterpenoids, and phenolic compounds are the major components of propolis (Bankova et al. 2000; Chen et al. 2008; Cursta-Rubio et al. 2007; Daugsch et al. 2008; Kumazawa et al. 2008; Markham et al. 1996; Popova et al. 2010). Some of these compounds are responsible for its biological activities (Bankova et al. 2000; Barros et al. 2007; Bassani-Silva et al. 2007; Bufalo et al. 2009; Cvek et al. 2007; Orsatti et al. 2010a; Orsatti et al. 2010b; Orsi et al. 2005; Zamami et al. 2007). There are three possible sources for the organic compounds of propolis: plants, secreted substances from honeybee metabolism, and materials that are introduced during propolis formation (Marcucci 1995). Propolis is typically composed of 50% resin and vegetable balsam, 30% wax, 10% essential and aromatic oils, 5% pollen and 5% other substances (Cirasino et al. 1987; Monti et al. 1983). Most of the studies on propolis composition and pharmacological effects have been performed on samples from Europe and Latin America (e.g. (Bankova et al. 2000; Daugsch et al. 2008; Barros et al. 2007; Monti et al. 1983)), whereas few have reported on propolis from north Africa (El-Hady & Hegazi 2002; Hegazi & El-Hady 2002) with none from Ethiopia. Ethiopia is located in north-eastern Africa with varied climatic and physiographic conditions that endowed the country with more than 7,000 species of flowering plants (Edwards 1976). They are considered as a potential for producing huge volume of propolis with high probabilities for various biologically active substances. However, many beekeepers in the country focus only on honey production.

Therefore, the main objective of this study is to determine the chemical compositions, characteristics and relative concentrations of organic compounds in the extractable organic matter of propolis samples collected from four different areas in Ethiopia.

## Results

**Figure 1:**
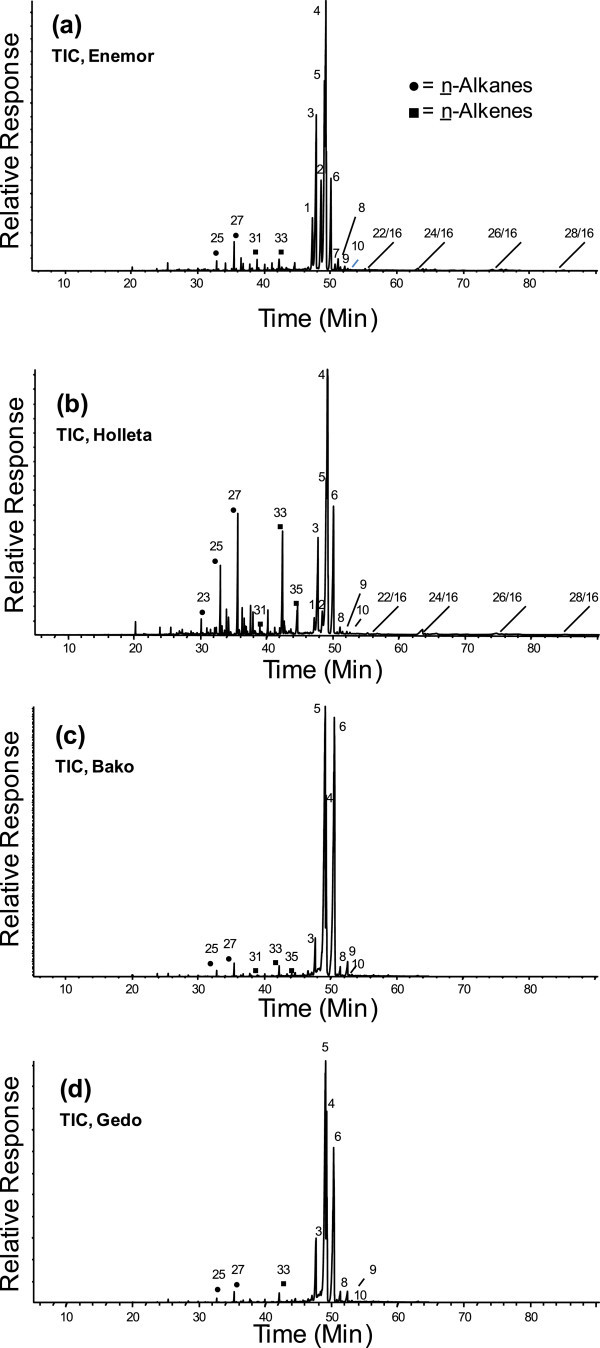
**Total ion current (TIC) traces showing the major organic tracers in propolis samples collected from.** **(a)** Enemor (Gurghe), **(b)** Holleta, **(c)** Bako, and **(d)** Gedo localities in Ethiopia (1 = β-amyrone, 2 = α-amyrone, 3 = β-amyrin, 4 = α-amyrin, 5 = β-amyryl acetate, 6 = α-amyryl acetate, 7 = lup-20(29)-en-3-one, 8 = lupeol, 9 = β-lupeyl acetate, 10 = α-lupeyl acetate, 22/16, 24/16, 26/16 and 28/16 are docosyl hexadecanoate, tetracosyl hexadecanoate, hexacosyl hexadecanoate and octacosyl hexadecanoate, respectively; numbers above the symbols indicate the carbon number).

**Figure 2:**
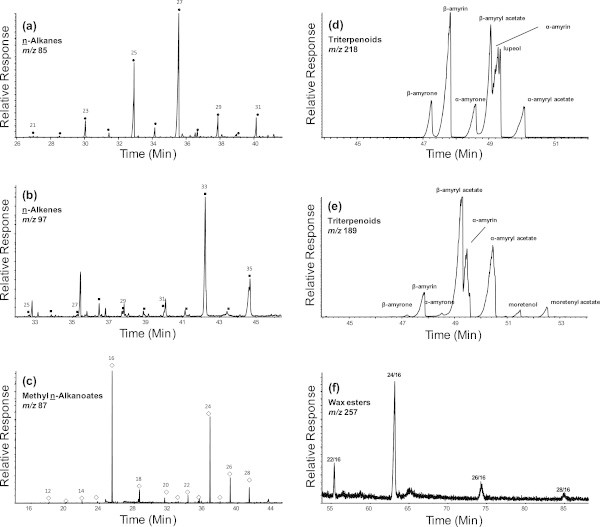
**Examples of typical GC-MS key ion plots for (a) n-alkanes, (b) n-alkenes, (c) methyl n-alkanoates, (d) and (e) triterpenoids and (f) wax esters (numbers refer to the carbon number)**.

The main features of the GC–MS data and the major extractable organic compound concentrations for the propolis samples are shown in Figure 1 and listed in Table 1, respectively. They included titerpenoids, *n*-alkanes, n-alkenes, *n*-alkanoic acids, methyl *n*-alkanoates, and long chain wax esters (Table 1). The major triterpenoids were α- and β-amyrins (0.0-83.8% of total extract), and α- and β-amyryl acetates (0.0-53.8% of total extract) (Figures 1, 2d,e and Table 1). For *n*-alkanes the major compounds were pentacosane (0.20-4.50% of total extract), heptacosane (0.44-7.56% of total extract), nonacosane (0.08-1.27% of total extract), and hentriacontane (0.06-1.16% of total extract) (Table 1, Figure 2a). Tritriacontene (0.53-9.06% of total extract) was the major compound for the n-alkene group (Table 1 and Figure 2b), whereas methyl hexanoate (0.09-0.30% of total extract) was the major compound for the methyl *n*-alkanoates (Table 1 and Figure 2c). The major compound for wax esters was found to be tetracosyl hexadecanoate (0.19-1.75%; Table 1 and Figure 2f).

**Table 1:** **The relative concentrations (%), and organic parameters of the various lipid compound groups of propolis samples from different regions of Ethiopia**

			Enemore	Holleta	Bako	Gedo	Average	SD
**Yield (%)**			27.2	33.4	62.0	64.2	46.7	19.1
**Compound**	**Composition**	**M.W.**						
**Triterpenoids**								
β-Amyrone	C_30_H_48_O	424	0.08	0.01	0.36	1.16		
α-Amyrone	C_30_H_48_O	424	0.19	0.01	0.00	1.88		
β-Amyrin	C_30_H_50_O	426	0.29	0.12	3.29	0.00		
α-Amyrin	C_30_H_50_O	426	83.79	63.11	0.00	3.16		
β-Amyryl acetate	C_32_H_52_O_2_	468	0.15	0.19	**44.88**	**53.79**		
α-Amyryl acetate	C_32_H_52_O_2_	468	0.00	0.00	**46.72**	29.72		
Lupeol	C_30_H_50_O	426	1.81	0.24	2.29	1.84		
Moretenol	C_32_H_52_O_2_	426	0.46	0.19	0.05	2.04		
Moretenyl acetate	C_32_H_52_O_2_	468	0.15	0.09	0.00	0.09		
**Total**			**86.91**	**63.96**	**97.60**	**93.67**	**85.53**	**15.04**
**n-Alkanes**								
Heneicosane	C_21_H_44_	296	0.00	0.00	0.00	0.01		
Docosane	C_22_H_46_	310	0.00	0.00	0.00	0.00		
Tricosane	C_23_H_48_	324	0.14	1.02	0.03	0.05		
Tetracosane	C_24_H_50_	338	0.01	0.25	0.01	0.02		
Pentacosane	C_25_H_52_	352	0.58	4.50	0.20	0.37		
Hexacosane	C_26_H_54_	366	0.10	0.58	0.03	0.04		
Heptacosane	C_27_H_56_	380	**1.88**	**7.56**	**0.44**	**0.93**		
Octacosane	C_28_H_58_	394	0.12	0.36	0.02	0.04		
Nonacosane	C_29_H_60_	408	0.38	1.27	0.08	0.25		
Triacontane	C_30_H_62_	422	0.02	0.18	0.00	0.03		
Hentriacontane	C_31_H_64_	436	0.32	1.16	0.06	0.21		
**Total**			**3.54**	**16.89**	**0.87**	**1.94**	**5.82**	**7.48**
CPI (o/e)^a^			13.56	10.95	12.26	14.25	12.75	1.46
**n-Alkenes**								
Pentacosene	C_25_H_50_	350				0.009		
Hexacosene	C_26_H_52_	364				0.012		
Heptacosene	C_27_H_54_	378				0.014		
Octacosene	C_28_H_56_	392	1.68	1.40	0.06	0.093		
Nonacosene	C_29_H_58_	406	0.27	0.17	0.02	0.033		
Triacontene	C_30_H_60_	420	1.31	0.83	0.04	0.042		
Hentriacontene	C_31_H_62_	434	0.05	1.27	0.01	0.037		
Dotriacontene	C_32_H_64_	448	0.89	0.48	0.04	0.047		
Tritriacontene	C_33_H_66_	462	8.11	9.06	0.53	0.912		
Tetratriacontene	C_34_H_68_	476	0.20	0.13	0.00	0.033		
Pentatriacontene	C_35_H_70_	490	0.86	2.54	0.14	0.284		
**Total**			**6.61**	**15.88**	**0.85**	**1.515**	**6.23**	**6.96**
CPI (o/e)^b^			2.49	5.39	6.10	7.24	5.30	**2.02**
**Methyl n-Alkanoates**								
Methyl dodenoate	C_13_H_26_O_2_	214	0.000	0.000	0.000	0.001		
Methyl tridecanoate	C_14_H_28_O_2_	228	0.000	0.000	0.000	0.000		
Methyl tetradecanoate	C_15_H_30_O_2_	242	0.004	0.004	0.001	0.001		
Methyl pentadecanoate	C_16_H_32_O_2_	256	0.003	0.001	0.001	0.001		
Methyl hexadecenoate	C_17_H_32_O_2_	286	0.006	0.000	0.000	0.000		
Methyl hexadecanoate	C_17_H_34_O_2_	270	0.304	0.222	0.093	0.123		
Methyl heptadecenoate	C_18_H_34_O_2_	282	0.001	0.000	0.000	0.001		
Methyl octadecenoate	C_19_H_36_O_2_	296	0.006	0.013	0.008	0.011		
Methyl octadecanoate	C_19_H_38_O_2_	298	0.029	0.021	0.008	0.012		
Methyl nonadecanoate	C_20_H_40_O_2_	312	0.001	0.000	0.000	0.000		
Methyl eicosanoate	C_21_H_42_O_2_	326	0.010	0.007	0.004	0.007		
Methyl heneicosanoate	C_22_H_44_O_2_	340	0.001	0.000	0.000	0.001		
Methyl docosanoate	C_23_H_46_O_2_	354	0.018	0.010	0.004	0.005		
Methyl tricosanoate	C_24_H_48_O_2_	368	0.031	0.004	0.002	0.002		
Methyl tetracosanoate	C_25_H_50_O_2_	382	0.199	0.160	0.045	0.065		
Methyl pentacosanoate	C_26_H_52_O_2_	396	0.001	0.000	0.000	0.001		
Methyl hexacosanoate	C_27_H_54_O_2_	410	0.058	0.048	0.013	0.017		
Methyl heptacosanoate	C_28_H_56_O_2_	424	0.000	0.000	0.000	0.000		
Methyl octacosanoate	C_29_H_58_O_2_	438	0.035	0.029	0.009	0.012		
**Total**			**0.709**	**0.521**	**0.189**	**0.260**	**0.419**	**0.239**
**CPI(o/e as esters)** ^**c**^			16.92	87.04	45.63	0.28	37.47	37.99
**Wax esters**								
Docosyl hexadecanoate	C_38_H_76_O_2_	564	0.15	0.17	0.068	0.167		
Tetracosyl hexadecanoate	C_40_H_80_O_2_	592	0.74	1.75	0.191	0.372		
Hexacosyl hexadecanoate	C_42_H_84_O_2_	620	0.20	0.16	0.032	0.052		
Octacosyl hexadecanoate	C_44_H_88_O_2_	648	trace	trace	0.00	0.00		
**Total**			**1.10**	**2.08**	**0.29**	**0.59**	**1.02**	**0.78**

## Discussion

The chemical compositions of propolis samples obviously vary between different samples (Popova et al. 2010; El-Hady & Hegazi 2002; Hegazi & El-Hady 2002; Edwards 1976; Popova et al. 2011; Trusheva et al. 2003). Recent studies have shown that diterpenoids were the major compounds in propolis samples from Greece and Sicily (Popova et al. 2010; Trusheva et al. 2003; Popova et al. 2009). The major components of Greek propolis consist of communic, cupressic, isocupressic acids and totarol (Popova et al. 2010), indicating a potential origin from conifer (e.g. cedar) resin (Cox et al. 2007). Triterpenoids including β-amyrin, β-amyrone, lupeol, and lupenone, and polyprenyl benzophenones such as 7-epi-nemorosone, 7-epi-clusianone, xanthochymol, and gambogenone have been detected in propolis samples from the Brazilian Amazon (de Castro Ishida et al. 2011). Propolis samples from Egypt contained caffeate esters, triterpenoids with major diterpenoids, but no aromatic acids and flavonoids (El-Hady & Hegazi 2002; Hegazi & El-Hady 2002). The results show that these propolis extracts include primarily lipid compounds from terrestrial plant sources as reported before (Bankova et al. 2000; Cursta-Rubio et al. 2007; Ugur et al. 2011; Campo Fernandez et al. 2008; Lotti et al. 2010; Melliou & Chinou 2004; Salatino et al. 2005). Phenols (e.g. flavonoids) or other antioxidants were not detected.

### Triterpenoids

Triterpenoids have been reported to occur in diverse plant species as resin or gum constituents (Cursta-Rubio et al. 2007; de Castro Ishida et al. 2011). They are rarely found in fungi and animals (Lutta et al. 2008). Therefore, the major source of triterpenoids is terrestrial vegetation (Hernández-Vázquez et al. 2010; Manguro et al. 2009; Moreau et al. 2009; Ramadan et al. 2009). They are found in plant leaves (Ramadan et al. 2009; van Maarseveen & Jetter 2009; Silva et al. 2009), bark (Hernández-Vázquez et al. 2010; Rosas-Acevedo et al. 2011; Feng et al. 2010; Vouffo et al. 2010), resins (Hernández-Vázquez et al. 2010; Manguro et al. 2009; Wang et al. 2011), and oils (Moreau et al. 2009; Akihisa et al. 2010; Bakowska-Barczak et al. 2009; Wesolowska et al. 2011). Their concentrations vary and depend on the plant species. For example, α- and β-amyrin are found in *Protium sp. Byrosonima fagifolia* and *Byrosonima crassifolia* (Hernández-Vázquez et al. 2010) and only α-amyrin is present in *Cassia obtusifolia* (Sob et al. 2010).

The main compounds in these propolis samples were triterpenoids. The relative concentrations of these substances ranged from 64.0% to 97.6% with a mean value of 85.5 ± 15.0%. They were mainly α- and β-amyrones, amyrins, and amyryl acetates, lupeol, and α- and β-lupeyl acetates. The highest triterpenoid concentrations were observed in the propolis from the Bako (97.6%) and Gedo (93.7%) areas, where the major vegetation is dominated by Acacia species, as well as Euphorbiaceae species (*Croton macrostachys*) and Boraginaceae species (*Cordia africana*). β-Amyrin was the major triterpenoid in the samples from the Enemor and Holleta with relative concentrations of 83.8% and 63.1%, respectively. Whereas, α-amyryl acetate was the major compound in the samples from the Bako with a relative concentration of 46.7%, followed by β-amyryl acetate at 44.9%. In the propolis from the Gedo the major compounds were also β-amyryl acetate (53.8%) followed by α-amyryl acetate (29.7%). Lupeol and α- and β- lupeyl acetates were also present in significant amounts (Table 1). This percentage variation in the contents is likely due to different plant species of the same family. As previously mentioned, these triterpenoid compounds were also detected in propolis samples from Brazil and Egypt (El-Hady & Hegazi 2002; Hegazi & El-Hady 2002; de Castro Ishida et al. 2011) as well as from Cuba (Márquez Hernández et al. 2010). This indicates that triterpenoid compounds are likely dominant components of propolis samples from tropical and semi-tropical regions.

The presence of triterpenoids (mainly amyrins and amyryl acetates) can act as antibacterial and antitumor agents (Sforcin & Bankova 2011; de Castro Ishida et al. 2011). Obviously, the main source of triterpenoids in propolis is the surrounding vegetation. Therefore, the determination of the chemical compositions of the regional vegetation should be considered, because it will be useful for investigating the pharmacologically active components of local plants as well as of propolis.

### *n*-Alkanes and *n*-alkenes

The relative concentrations of *n*-alkanes in these samples ranged from 0.87% to 16.9% of the total extracts with a mean of 5.82 ± 7.48% (Table 1). The lowest relative concentration (0.87%) was measured in the propolis from Bako, while the highest concentration (16.9%) was in the sample from the Holleta area. The dominant *n*-alkanes were in the range of C_21_ to C_31_, with a carbon number maximum concentration (C_max_) at 27 (e.g. Figure 2a, (Mazurek & Simoneit 1984)). The carbon preference index (CPI, (Mazurek & Simoneit 1984)) varied from 10.95 to 13.56 with an average of 12.75 ± 1.46 (Table 1). Plant wax *n*-alkanes generally have a C_max_ in the range of C_25_–C_31_, which varies depending on the plant species as well as the season and locality (e.g. (Eglinton & Hamilton 1967)). Thus, the odd carbon number preference of the C_21_-C_31_ n-alkanes and the C_max_ at 27 indicate the major sources of these n-alkanes are likely from the beeswax (Tulloch 1970).

The relative concentrations of the n-alkenes ( **Δ**^**1**^**or Δ**^**9**^) ranged from 0.85% to 15.92% with a mean of 6.23 ± 6.96%. The highest relative concentration (15.92%) was found in the propolis sample from the Holleta area and the minimum (0.85%) in the samples from Bako. The n-alkenes ranged from C_25_ to C_36_ with a C_max_ at 33. The odd carbon numbered *n*-alkenes were dominant with a CPI of 2.49 to 7.24 (mean 5.30 ± 2.02). The distribution of *n*-alkenes with major concentrations of the odd numbered homologues and C_max_ at 33 supports an origin from insect wax (Jackson 1972; Jackson & Baker 1970), possibly from alteration of long chain n-alkanols.

### Methyl *n*-alkanoates

The concentrations of methyl *n*-alkanoates were relatively low at 0.19% to 1.14% with a mean of 0.64 ± 0.40% (Table 1). They ranged from C_13_ to C_29_ with a C_max_ at 17 and 25 (as acids C_max_ = 16 and 24) (Figure 2c). Methyl *n*-alkanoates may be natural or form by transesterification of *n*-alkanoic acids during extraction as indicated by their low relative concentrations. The highest concentration (1.14%) was found for the propolis sample from Gedo and the lowest (0.19%) from Bako. The methyl *n*-alkanoates of these samples have a strong even carbon number predominance as the alkanoic acids (CPI > 17, except for Gedo, Table 1), indicating that they are originally from natural biota (Harwood & Russell 1984).

### Long chain wax esters

Long chain wax esters were also detected in these samples with relative concentrations of 0.29% to 2.08%, and consisting mainly of docosanyl-, tetracosanyl-, hexacosanyl- and octacosanyl hexadecanoates. The major compound of the wax esters was tetracosanyl hexadecanoate in all samples (Table 1, Figure 2f). They are likely derived from lipid components of terrestrial plants (Baker 1982; Kolattukudy 1976; Hamilton 1995) of the region or from waxes secreted by the bees (Tulloch 1971). Subsequent reports have shown that the components of waxes in some younger plants are generally alcohols (40%) and they are mainly wax esters (42%) in older plants (Avato et al. 1990; Bianchi et al. 1989). The vegetation wax ester composition depends not only on plant species, but also on the geographical location (Sforcin & Bankova 2011). Waxes secreted by bees contain more than 15% of wax esters (Katzav-Gozansky et al. 1997). Bee wax esters generally include tetradecyl-dodecanoate, tetradecanoate and hexadecanoate, as well as hexadecyl-tetradecanoate and hexadecanoate (Katzav-Gozansky et al. 1997).

### Unique composition

It has been reported that propolis components, which are complex, have biological properties including antimicrobial, antioxidant and anticancer activities (Lustosa et al. 2008; Naito et al. 2007; Diaz-Carballo et al. 2008). Propolis was also reported to have effects against cariogenic bacteria (de Castro Ishida et al. 2011). Triterpenoids are major and to date unique components of these propolis samples from different regions in Ethiopia, indicating a high potential as sources of biologically active substances. Further studies are needed to investigate the biological activities of these propolis samples, and the correlations between their chemical compositions and botanical origins.

## Conclusion

The solvent-extractable organic matter (DCM:MeOH) of propolis samples from four regions in Ethiopia have been characterized using GC–MS techniques. The mixed solvent was used to extract both polar and non-polar compounds of proplis samples. The major compounds were in order: triterpenoids > > *n*-alkanes ~ *n*-alkenes > long chain wax esters > methyl *n*-alkanoates. The predominant triterpenoids were α- and β-amyrins, α- and β-amyryl acetates, followed by lupeol, and α- and β-lupeyl acetates. *n*-Alkanes and n-alkenes ranged from C_21_ to C_31_ and C_25_ to C_35_ with C_max_ at 27 and 33, respectively. Long chain wax esters and methyl n-alkanoates were minor components in these samples. The sources of the major triterpenoids are from the regional Acacia waxes and gums. Phenols (e.g. flavonoids) or other antioxidants were not detectable in these samples.

The variation in the identities of propolis components among various reports is likely due to diverse environmental source vegetation, and different extraction methods and solvents used. Therefore, a standardized analytical method should be adopted in order to be able to compare results obtained by different investigators.

## Materials and methods

### Sampling

**Figure 3:**
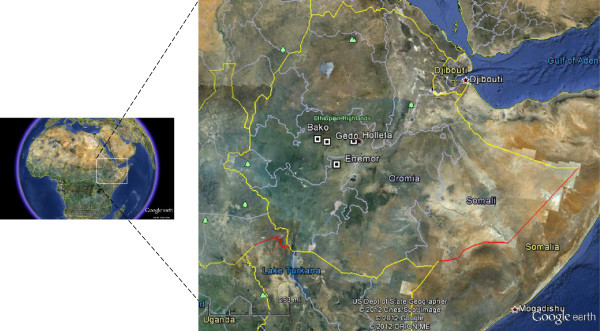
**Map showing the locations of the propolis sample collection.**

The propolis samples were collected from the central parts of Ethiopia representing highlands and midland areas. The specific areas were: Enemor (8°05’44.15”N; 37°52’06.15”E, at an attitude of 2000 m), Holleta (9°03’26.19”N, 38°33’22.45”E, altitude 2370 m), Bako (9°06’59.23”N, 37°03’23.02”E, altitude 1670 m), and Gedo (9°00’59.12”N, 37°26’58.19”E, altitude 2515 m) (Figure 3). The major vegetation of these regions is comprised of different species of Acacia, Euphorbiaceae sp. *Croton macrostachys*, and Boraginaceae sp. *Cordia africana.* The propolis samples were collected using a stainless steal spatula (>30 g of each) in a Teflon-caped glass container, labeled and kept in a freezer until analysis.

### Extraction

About 20 g of each sample was broken up and extracted three times using ultrasonic agitation for a 15 min period each with a mixture of dichloromethane (DCM) and methanol (MeOH, 40 mL, 3:1 v:v) mixture to make certain that both polar and non-polar compounds were extracted. The extraction was carried out in a precleaned beaker. The extract was then filtered using a filtration unit containing an annealed glass fiber filter for the removal of undissolved particles. The filtrate was first concentrated on a rotary evaporator and then reduced using a stream of dry nitrogen gas to a volume of approximately 2 mL. The volume was then adjusted to exactly 2 mL by addition of DCM:MeOH (3:1, v:v). A 50-μL aliquot of each total extract was derivatized with silylating reagent [N,O-bis(trimethylsilyl)trifluoroacetamide, BSTFA, Pierce Chemical Co.] by the standard procedure (Knapp 1979), before analysis by gas chromatography–mass spectrometry (GC–MS). This derivatizing agent replaces the H in hydroxyl groups with a trimethylsilyl [(CH_3_)_3_Si, i.e. TMS] group for better GC resolution of polar compounds.

### Chemical analysis

Instrumental analysis by GC–MS was carried out with an Agilent 6890 gas chromatograph coupled to a 5973 Mass Selective Detector, using a DB-5MS (Agilent) fused silica capillary column (30 m × 0.25 mm i.d., 0.25 μm film thickness) and helium as carrier gas. The GC was temperature programmed from 65°C (2 min initial time) to 310°C at 6°C min^−1^ (isothermal for 55 min final time) and the MS was operated in the electron impact mode at 70 eV ion source energy. Mass spectrometric data were acquired and processed using the GC–MS ChemStation data system.

### Identification and quantification

The identification of *n*-alkanes was based on the GC–MS data. Retention times were compared with those of external standards. The identities of triterpenoids, *n*-alkanes, *n*-alkenes, *n*-alkanoic acids, methyl *n*-alkanoates, and long chain wax esters are based primarily on their mass spectra (i.e. key ions at *m/z* 191/189, 85, 83, 117, 87, and 257, respectively), comparison with those of standards or in the literature, and gas chromatographic retention times. Average response factors were calculated for each compound. All quantifications were based on the compound peak areas derived from the ion fragmentograms correlated with the total ion current (TIC) trace.
